# Complete Genome Sequence of *Pasteurella multocida* Serotype A Strain PMTB2.1 Isolated from Buffaloes That Died of Septicemia in Malaysia

**DOI:** 10.1128/genomeA.01190-17

**Published:** 2017-11-02

**Authors:** Shagufta Jabeen, Yap Huan Yong, Faez Jesse Firdaus Abdullah, Zunita Zakaria, Nurulfiza Mat Isa, Yung-Chie Tan, Wai-Yan Yee, Abdul Rahman Omar

**Affiliations:** a Institute of Bioscience, Universiti Putra Malaysia, Serdang, Selangor, Malaysia; b Faculty of Veterinary Medicine, Universiti Putra Malaysia, Serdang, Selangor, Malaysia; c Faculty of Biotechnology and Biomolecular Sciences, Universiti Putra Malaysia, Serdang, Selangor, Malaysia; d Institute of Tropical Agriculture and Food Security, Universiti Putra Malaysia, Serdang, Selangor, Malaysia; e Institute of Microbiology, University of Sindh Jamshore, Hyderabad, Sindh, Pakistan; f Codon Genomics, Taman Dutamas Balakong, Seri Kembangan, Selangor, Malaysia

## Abstract

*Pasteurella multocida* causes pneumonic pasteurellosis and hemorrhagic septicemia (HS) in large ruminants. In this study, we determined the complete genome sequence of* P. multocida* strain PMTB2.1 capsular serotype A isolated from buffaloes that died of septicemia.

## GENOME ANNOUNCEMENT

*Pasteurella multocida* is the causative agent of a wide-spectrum disease manifested by pneumonic pasteurellosis and hemorrhagic septicemia (HS) in buffalo and cattle (1). *P. multocida* serotype A has been reported to cause fatal pneumonia and septicemia (2). Neither the complete genome sequence of *P. multocida* serotype A from Malaysia nor the complete genome of *P. multocida* serotype A sequenced using third-generation sequencing technology has been previously published. Furthermore, *P. multocida* virulence genes and their interactions in influencing the virulence of the bacteria and their pathogenicity in infected animals are poorly characterized.

The *P. multocida* strain PMTB2.1 was isolated from buffaloes that died of septicemia in Peninsular Malaysia. The genomic DNA of PMTB2.1 was extracted using the DNeasy tissue kit (Qiagen, Germany) and identified based on species-specific PCR, as previously described (3). PMTB2.1 DNA genome sequencing was performed using the Pacific Biosciences RS II sequencing platform (4).

A total of 49,844 circular consensus sequence (CCS) reads (89,242,957 bp) and 523,361 continuous long reads (CLR) (899,097,638 bp) were extracted from the raw sequencing data. Error correction of the CLR based on the CCS reads was carried out by the module PacBio ToCA of Whole-Genome Shotgun Assembler v8.2, which generated 13,231 reads with a mean read length of 2,000 bp and 26× coverage. The genome was assembled (*de novo*) by runCA (Celera Assembler) using default parameters, which generated 3 contigs with sizes of 1,057,336 bp, 946,414 bp, and 338,900 bp, respectively, and a total length of 2,342,650 bp. The gaps between contigs were resolved with PCR sequencing using the primer-walking strategy, whereas the circular chromosome was confirmed by PCR. The genome was annotated using the NCBI Prokaryotic Genomes Automatic Annotation Pipeline (PGAAP). Finally, the complete genome sequence of *P. multocida* strain PMTB2.1, with a genomic size of 2,315,138 bp and a GC content of approximately 40.32%, was produced as a single contiguous circular chromosome. The PMTB2.1 genome sequence contains 2,176 potential genes, 33 of which were likely to be nonfunctional due to the presence of either frameshift mutations (29 genes) or internal stop codons (4 genes). The genome sequences encode 2,097 DNA coding sequences (CDSs) and 79 RNAs (rRNA, tRNA, and noncoding RNA [ncRNA]) genes. Characterization of the complete genome sequences is an important step in further investigation of the genomic structure and comparative pathogenomic characteristics of the bacteria.

### Accession number(s).

The complete genome sequences of *P. multocida* strain PMTB2.1 were submitted to the NCBI GenBank database under the accession no. CP007205. 
